# Phytochemicals, Pharmacological Effects and Molecular Mechanisms of Mulberry

**DOI:** 10.3390/foods11081170

**Published:** 2022-04-18

**Authors:** Junyu Hao, Yufang Gao, Jiabao Xue, Yunyun Yang, Jinjin Yin, Tao Wu, Min Zhang

**Affiliations:** 1 State Key Laboratory of Food Nutrition and Safety, Food Biotechnology Engineering Research Center of Ministry of Education, College of Food Science and Engineering, Tianjin University of Science & Technology, Tianjin 300457, China; haojunyu05@163.com (J.H.); x2904277148@126.com (J.X.); yinjinjin@tust.edu.cn (J.Y.); zm0102@sina.com (M.Z.); 2 National Engineering Laboratory of Intelligent Food Technology and Equipment, College of Biosystems Engineering and Food Science, Zhejiang University, Hangzhou 310058, China; gaoyufang@zju.edu.cn; 3 College of Life Sciences, China Jiliang University, Hangzhou 310018, China; yangyunyun@zju.edu.cn; 4 College of Food Science and Bioengineering, Tianjin Agricultural University, Tianjin 300384, China

**Keywords:** mulberry, composition, anthocyanins, flavonoids, biological activities

## Abstract

There are numerous varieties of mulberry, and each has high medicinal value and is regarded as a promising source of traditional medicines and functional foods. Nevertheless, the nutrients and uses of mulberry differ from species (*Morus alba* L., *Morus nigra* L. and *Morus rubra* L.). Phenolic compounds are prominent among the biologically active ingredients in mulberry, especially flavonoids, anthocyanins and phenolic acids. Epidemiologic studies suggest that mulberry contains a rich, effective chemical composition and a wide range of biological activity, such as antioxidant, anti-inflammatory, anti-tumor and so on. However, compared with other berries, there has been a lack of systematic research on mulberry, and this hinders its further expansion as a functional fruit. The main purpose of this review is to provide the latest data regarding the effective chemical constituents and pharmacological effects of mulberry to support its further therapeutic potential and health functions.

## 1. Introduction

Mulberry, which belongs to the genus Morus of the Moraceae family, is an aggregated berry that is oval-shaped, rich in nutrition, sweet and soft, with a unique flavor [1,2]. Mulberry is distributed in east, west and southeast Asia, southern Europe, southern North America, northwestern South America and some areas of Africa [3]. There are 24 species of Morus and one subspecies, with at least 100 known varieties [4]. Studies have shown that mulberry is beneficial for human health, which may be related to the compounds it contains, such as phenols, amino acids and sugars [5,6]. Since ancient times, mulberries have been used as fruits and herbs and have been listed by the Chinese Ministry of Health as one of the “food and medicine” agricultural products. The fruit is a high-quality natural raw material that is used for the production of modern food and diet regimens. It is commonly eaten, often dried, or processed into wine, syrups, canned food, fruit juice, jam and beverages [3,4,7,8,9,10]. The anthocyanin content in mulberry wine is much higher than that of red wine, and regular consumption can increase immunity.

There are many varieties of mulberry, with the most common species consisting of black mulberry (*Morus nigra* L.), white mulberry (*Morus alba* L.) and red mulberry (*Morus rubra* L.) [11]. Some studies have revealed that black mulberry has a higher content of total phenolics, total flavonoids, total anthocyanins and more antioxidant compounds than red mulberry or white mulberry [4,12,13]. Some authors point out that the nutrient and plant chemistry of mulberry are closely related to the area in which it was cultivated [14,15,16].

At present, a limited number of reviews on the phytochemical and pharmacological properties of mulberry have been published. The current review attempted to provide holistic insight into the composition of mulberry, which would promote human health, and to investigate biological activities against chronic diseases.

## 2. Composition of Mulberry

Mulberry is rich in nutrients, approximately 0.5–1.4% protein and about 7.8–9% carbohydrates [17]. Mulberry contains neutral sugars such as arabinose, galactose, glucose, rhamnose, xylose, mannose and also contains a large amount of uronic acid, namely in the form of galacturonic acid and glucuronic acid [18,19,20,21]. The most abundant amino acid in mulberry is glutamate, which accounts for approximately 20%, followed by glycine and aspartate [22]. It also contains lysine, leucine, isoleucine, histidine, threonine, tryptophan and glycine, among others. Among these amino acids, leucine, threonine, isoleucine, glycine, threonine, valine, tryptophan, arginine, aspartic acid and serine are found in a higher content in white mulberry when compared with black mulberry. In contrast, the content of lysine, histidine, and proline are higher in black mulberry and lower in white mulberry [23]. The fat content of mulberry is extremely low, and linoleic acid, oleic acid, palmitic acid and stearic acid make up 69.66–78.02% of the total fatty acids [24]. The vitamins in mulberry are mainly vitamin C, vitamin A and some B groups [16]. The organic acids of mulberry are succinic acid, acetic acid, malic acid, citric acid and tartaric acid [3]. The content of titrable acid is 0.20–2.65%, and the content in black mulberry is higher than that in white mulberry [4,23,25,26,27]. The minerals in mulberry are potassium, calcium, phosphorus, sodium, zinc, copper and selenium. Studies have shown that the content of potassium in black mulberry is much higher than that in other fruits [13]. Soluble solids mainly include sugar, acid, vitamins and minerals, and their content can directly affect the taste of fruits and vegetables. The total soluble solid content of mulberry is 6.2–25.8% [4,23,25,26].

Mulberry contains many phenolic compounds, and Table 1 contains the results of the determination of total phenol and total flavonoids from recent years. In addition to the above ingredients, mulberry also contains alkaloid compounds (quinine, 1-deoxynojirimycin) and α-glucosidase inhibitors [28,29,30]. Different varieties of mulberry possess different chemical compositions and nutritional statuses, which are related to the climate, topography and soil conditions [7].

## 3. Content of Phenolic Compounds

Polyphenols play an important role in promoting human health and are the most relevant family of phytochemicals [53].

### 3.1. Flavonoids

Among them, flavonoids constitute a very wide range of groups and are distributed in a variety of vegetables and fruits. They have a common basic structure: C6-C3-C6, which usually forms an oxygen-containing heterocycle. Flavonoids are usually associated with sugars (glycosides), and therefore, they tend to be water-soluble.

### 3.2. Anthocyanins

Mulberries are especially rich in flavonoids, specifically anthocyanin [4,31,32,54,55]. Anthocyanin is the main active ingredient and chromogenic substance of mulberry, which is why mulberry is considered to be an important source of anthocyanin in the diet [33]. Its anthocyanin content is high, pigment is stable, can dissolve completely in water and has increased bioactive activity; therefore, it becomes a fruit pigment that cannot be replaced. Mulberry is an optimal source for extracting anthocyanin from pine bark, pine needles and grape seeds. Therefore, together with sea buckthorn, it is listed as a functional health food with the international development of the third generation of “fruit resources”. The most abundant anthocyanin in mulberry is cyanidin-3-glucoside (C3G), representing 53.94–78.23% of the total anthocyanins; cyanidin-3-rutinoside (C3R) accounts for 19–43.83%, and pelargonidin-3-glucoside (P3G) is measured in a proportion close to 5% [34,56,57,58,59,60]. The content of mulberry anthocyanins is shown in Table 2; white varieties do not contain any anthocyanins [24,25]. The content of anthocyanin in mulberry is related to mulberry variety, maturity, climate, soil, pruning of mulberry trees, pest control and other factors [3,7,61].

### 3.3. Phenolic Acids

Mulberries contain phenolic acids (chlorogenic acid, gallic acid, protocatechuic acid, p-coumaric acid, O-coumaric acid, ferulic acid, caffeic acid, and vanillic acid), rutin, quercetin and resveratrol [3,15,56,66,67]. The main phenolic acids in mulberry are hydroxycinnamic acid derivatives [25]. Gallic acid and protocatechuic acid are the main derivatives of hydroxybenzoic acid in black mulberry [35,66]. The content of phenolic acids in mulberry is 0.02952–0.17564 mg/g fw [68]. Chlorogenic acid is the main phenolic component in black mulberry, while ruin is the dominant phenolic in white and red mulberry [3]. Chlorogenic acid is the main acid in the sugarless extract of black and white mulberry [36]. Gundogdu et al. described the concentration range of chloric acid in mulberry as 0.119–3.106 mg/g fw [3]. Butkhup et al. found that its concentration in white mulberry was 0.01 to 0.06 mg/g dw [35], and the concentration range of chlorogenic acid in white mulberry and black mulberry was reported by Sanchez-Salcedo et al. as 0.15–0.97 mg/g dw and 0.35–3.18 mg/g dw [13], respectively. The amount of chlorogenic acid in black mulberry was higher than that in white mulberry. As mulberry matures, the amount of chlorogenic acid and its isomers gradually decreases [69]. Song et al. detected resveratrol in 38 mulberry varieties from China at 0.0021–0.0053 mg/g [52]. Chon et al. found a significant difference in the total phenol content measured by different solvents [37]. The content of total phenolic compounds in mulberry is shown in Table 1, with black mulberry containing a higher amount than white mulberry [12,38].

## 4. Beneficial Effects of Mulberry

Recent studies have revealed that mulberries have positive biological activities against chronic diseases such as cancer, neurotoxicity, obesity, diabetes and memory degradation, among others. These protective properties are related to the potent antioxidant and anti-inflammatory activities of polysaccharides [70,71,72,73,74], carotenoids [75,76], rutin, resveratrol [77,78,79], anthocyanins [56,80], minerals (Se, Fe, Zn, Cr) [15], glutamate and other phenolic compounds [70,81]. Oxidative stress in the body produces free radicals, and phenolic compounds can protect the body from free radical-induced side effects [82,83]. Mulberry polysaccharide can regulate immune function, as it has excellent reducing power and hydroxyl radical scavenging capacity. The free radical clearance rate of mulberry polysaccharides is better than that of ascorbic acid and rutin, and the IC50 is 0.059–0.119 mg/mL [39,84]. The digestive rate of mulberry polysaccharides in gastric juice is the fastest and is second in intestinal fluid; saliva is not effective for breaking down these polysaccharides [25]. Vitamin A plays an important role in normal growth and tissue repair of the human body [14]. It is important to maintain the normal physiological function of the visual system and immune system. Carotenoid is an important dietary source of vitamin A [85]. Rutin is an antioxidant that has been shown to have anti-inflammatory properties and is essentially a flavonoid glycoside [86]. The content of rutin in mulberry is higher than in strawberry [67]. Rutin, morin, quercetin and myricetin are the main flavonols in mulberry [15], which are reported to be effective antioxidants [87]. Anthocyanin is a flavonoid that is found in large quantities in fruits and vegetables. However, the bioavailability of anthocyanin in the body is low. Studies have shown that anthocyanin absorbed into the plasma accounts for only 1% of the total intake, which may be low because of limited intestinal absorption or due to the high cellular uptake and excretion rate of anthocyanin [88,89]. Some authors point out that the observed biological effect may not originate from the flavonoid itself, but rather, its secondary metabolites, because the flavonoid is detected in its original form in very low quantities [90].

### 4.1. Anti-Oxidant Activity

Oxidative stress reflects the imbalance of the body’s peroxidation and anti-oxidation. On the one hand, it is the systemic manifestation of reactive oxygen species (ROS). On the other hand, it involves the repair of the detoxification and oxidative stress damage caused by the organism’s peroxidation intermediates. The anti-oxidation ability of mulberry is particularly prominent [91,92,93]. This fact explains why it can slow down the aging process and prevent three primary human killers: cardiovascular disease, cancer and diabetes. The antioxidant capacity of mulberry has been repeatedly verified. Xu et al. used isolated compounds from mulberry to measure oxygen radical absorbance capacity (ORAC) and DPPH radical scavenging assay; all compounds showed potent ORAC with higher ORAC values than the positive control [5,94,95]. Zhang et al. used 700 μmol/L H_2_O_2_ to induce PC-12 cells for 8 h to establish a cell oxidative injury model. The high concentration of mulberry polysaccharide component T3-3 was found to have extremely strong cellular anti-oxidation effects, which resulted in an increase in cell viability of 41.81% [71]. Chang et al. found that mulberry extract and mulberry anthocyanin extract both scavenge free radicals, inhibit low-density lipoprotein oxidation and reduce atherosclerosis caused by macrophages. The latter was 10 times better than the former [96,97,98].

The molecular mechanism of this protection is not clear. However, it has been suggested that mulberry may participate in the insulin signaling pathway, regulate various transcription factors, improve the enzymatic activity of superoxide dismutase (SOD), glutathione peroxidase (GSH-Px) and catalase (CAT) [21,99] and reduce the concentration of malondialdehyde (MDA), blood urea nitrogen (BUN), serum creatinine (Scr), nitric oxide (NO) and thiobarbituric acid-related substances (TBARS) [15,100]. Mulberry decreases the dysfunction of diabetic mice through the 5′ adenosine monophosphate-activated protein kinase (AMPK)/acetyl-CoA carboxylase (ACC)/mechanistic target of the rapamycin (mTOR) pathway. It promotes the phosphorylation of AMPK in insulin-sensitive tissues, inhibits the expression of ACC and mTOR, changes the expression of p38-mitogen-activated protein kinase (MAPK) and peroxisome proliferator-activated receptor gamma coactivator-1 alpha (PGC-1α) and protects hepatocytes against oxidative stress [40]. Luteolin hexoside and luteolin rutinoside clear 1,1-diphenyl-2-picrylhydrazyl (DPPH) and 2, 2′-azino-bis (3-ethylbenzothiazoline-6-sulfonic acid) (ABTS) as well as inhibit α-glucosidase and show a significant positive correlation with each other [38]. Anthocyanins can inhibit lipid peroxidation, improve the thermal stability of DNA’s three-dimensional structure and form complexes to protect DNA from oxidative damage [101,102,103]. Following the most recent research, Table 3 describes the main biological effects attributed to anthocyanins. The specific antioxidant capacity of mulberry is shown in Table 4.

Studies have shown that the ability of mulberry to resist lipid oxidation increases with concentration, with 23.7–47.6% at 76 μg and 52.7–73.3% at 255 μg [39]. Studies have shown that the DPPH scavenging power of mulberry increased with maturity, according to the order of fully-ripened (49.83%) > semi-ripened (43.76%) > unripened (31.74%). The DPPH scavenging power of white mulberry is higher than that of other mulberry varieties [27,61]. However, generally speaking, the total antioxidant activity of black mulberry is stronger [125]. The total antioxidant activity of the sugar-free extracts of black mulberry ranged from 1.19 to 1.25 mmol Trolox/g, and white mulberry ranged from 0.75 to 0.78 mmol Trolox/g [126].

### 4.2. Hypoglycemic Activity and Hyperlipidemia Action

Mulberry can effectively regulate blood lipids and has certain protective effects on the cardiovascular and cerebrovascular systems [15]. Mulberry can improve the antioxidant status of the blood and liver and weaken lipid peroxidation. Mulberry polysaccharide significantly inhibits the content of low-density lipoprotein-C (LDL-C), decreases triglyceride and total cholesterol in the serum and liver of rats fed a high-fat diet, reduces the atherogenic index and increases serum high-density lipoprotein-C (HDL-C) levels [6]. In experiments simulating in vitro digestion, mulberry polysaccharide inhibits lipid digestion, and this effect is positively correlated. It is mainly related to gastric fluid and intestinal fluid, and saliva is not effective for the digestion of mulberry [18]. Choi et al. found that water-soluble polysaccharide JS-MP-1 in mulberry can reduce the number of adipocytes by inhibiting the proliferation of preadipocytes [19]. Mulberry anthocyanin can reduce GLU, reduce leptin secretion, regulate fat production and lipolysis, decrease the size of fat cells and inhibit lipid accumulation [99,118,119,127]. Chang et al. found that mulberry anthocyanin was beneficial to the expression of PPARα and carnitine palmitoyl transferase 1 (CPT1), phosphorylation of AMPK and fatty acid oxidation and inhibited the synthesis of fatty acids and the accumulation of oleic acid-induced lipids in HepG2 human hepatoma cells [96,128]. Mulberry has a significant inhibitory effect on blood glucose in diabetic mice [36,106]. Mulberry anthocyanin prevents pancreatic islet degeneration and reduces insulin resistance in HepG2 cells [106,127]. C3G inhibits the apoptosis of pancreatic cells caused by glucose and oxidative stress [107]. Mulberry anthocyanin treats diabetes through AMPK, and its mechanism of action is similar to that of metformin, a drug for diabetes; its therapeutic effect is affected by PGC-1α [40].

### 4.3. Anti-Inflammatory Activity

Inflammatory disease is a complex process involving multiple cells, and its basic mechanism is the defense response of the body to protect and repair the damage of external inflammatory factors. Mulberry extract has good anti-inflammatory effects, although the mechanism is not particularly clear. It may have an important relationship with the promotion of the activation of murine macrophage RAW264.7 and the production of inflammatory factors [110]. JS-MP-1, a water-soluble polysaccharide isolated from Korean mulberry fruits, stimulates the RAW 264.7 cell to release RANTES, macrophage inflammatory protein-α (MIP-1α), tumor necrosis factor-α (TNF-α), and interleukin-6 (IL-6) [20,111]. The former two can attract leukocytes to the reaction site, and the latter two can mediate a variety of immune responses. Using lipopolysaccharide (LPS) to stimulate RAW264.7 cells to establish a model of inflammation, Zhu et al. found that mulberry could inhibit the secretion of nitric oxide (NO), prostaglandin E2 (PGE2), other inflammatory factors (in a dose-dependent manner) and the expression of inducible nitric oxide synthase (iNOS) and cyclooxygenase-2 (COX-2), which may be related to the high resveratrol content in mulberry [129]. Wu et al. found that mulberry anthocyanin inhibited the expression of TNFα, IL-6, iNOS and nuclear factor-kappa light-chain-enhancer of activated B cells (NF-кB) and also inhibited the oxidative stress and inflammation caused by diet [99]. Mulberry is rich in rutin and quercetin, which have a strong immunoregulatory effect on spleen cells. They can reduce the secretion ratio of T-helper cells 1/2 (Th1/Th2) and pro-inflammatory/anti-inflammatory cytokines, which results in the anti-inflammatory effect of mulberry being preventive, not therapeutic [57,130]. Yan et al. found that the anti-inflammatory effect of mulberry was related to the improvement of metabolic disorders related to obesity, which was beneficial to messenger ribonucleic acid (mRNA), expression of insulin receptor substrate 1 (IRS1), sterol regulatory element-binding protein-1c (SREBP-1c) and peroxisome proliferator-activated receptor-gamma coactivator-1α (PGC-1α) [40].

### 4.4. Anti-Tumor and Anti-Cancer Activity

In 1982, the U.S. National Academy of Sciences published “Diet, Nutrition, and Cancer”, which emphasized the importance of fruits and vegetables in the diet. In particular, citrus, crucifer and other fruits and vegetables rich in carotene have been shown to be effective in preventing cancer [131]. The U.S. National Research Council and the National Cancer Institute recommend eating more than five servings of fruits and vegetables per person per day to reduce the risk of cancer and heart disease [132,133]. The anticancer effect of mulberry has been confirmed in various cell lines, which have shown the inhibition of cancer cell growth and induction of apoptosis [112,134]. Cheng et al. found that mulberry polyphenol extracts (MPE) induce autophagy in Hep3B cells by inhibiting Akt and mTOR phosphorylation [135]. Zheng et al. confirmed that mulberry can indirectly enhance the immune function of mice to inhibit the formation of colon cancer [113]. Yan et al. confirmed that mulberry anthocyanin can regulate glucose metabolism of hepatocellular carcinoma cells by promoting glycogen synthesis and reducing the production of glucose [108]. Liu et al. detected that anthocyanin in mulberry had a significant inhibitory effect on the development of gastric cancer. A possible mechanism is to increase the ratio of LC3-II/LC3-I and BAX/BCL-2 in gastric cancer SGC-7901 cells and promote the expression of Beclin1, Caspase-8. Huang and other researchers have shown that mulberry anthocyanin can effectively inhibit the metastasis of melanoma, which may be related to the Ras/PI3K signaling pathway [114]. Nie et al. found that mulberry anthocyanin can inhibit DNA synthesis by blocking the cell cycle in the S phase so that the tumor cells cannot undergo normal mitosis [136]. Long et al. discovered that mulberry anthocyanins could serve as a novel therapy method for thyroid cancer mainly by inducing apoptosis and autophagic-induced cell death [115]. Recent studies found that mulberry anthocyanins can induce apoptosis and enhance the autophagy of cancer cells; this can be used as a new treatment for cancer cells. Mulberry anthocyanin exhibits anticancer and anti-tumor effects in a dose-dependent manner [116,136].

### 4.5. Anti-Bacterial and Anti-Viral Activity

It is known that mulberry inhibits the proliferation and growth of many bacteria, including *Escherichia coli*, *Bacillus*, and *Staphylococcus aureus*. Mulberry red pigment has the dual function of pigmentation and bacteriostasis, which is an ideal functional for natural pigment. It has a strong inhibitory effect on *E. coli* and a weaker inhibitory effect on *S. aureus*, *Streptococcus mutans*, and *Bacillus subtilis*, while it has almost no inhibitory effect on molds and yeasts. Its antibacterial capacity is proportional to the concentration and inversely proportional to the pH of the environmental medium [98,137]. The flavonoids in mulberry have a greater inhibitory effect on bacteria (*E. coli* and *S. aureus*) than on molds (*Aspergillus niger* and *Penicillium citrinum*), and the antibacterial activity against *S. aureus* is slightly stronger in both bacteria [138]. Resveratrol exists in two forms: free form and glycoside binding state. Both forms exist in the cis and trans configuration and have neuroprotective and antioxidative effects, and therefore, have potential antiviral and immunomodulatory effects [77,78]. Anthocyanin may inhibit the activity of extracellular microbial enzymes, destabilize the plasma membrane and deprive the microbes of the substrates necessary for growth, thus affecting microbial metabolism [41,139]. Gram-positive bacteria are usually more susceptible to anthocyanin than Gram-negative bacteria [140].

Compared with other germicidal agents, mulberry has a great advantage and is safe for intestinal flora. The antiviral activity of mulberry shows that it is not only a promising fruit, but also a potential therapeutic drug.

### 4.6. Hepatoprotective and Renoprotective Activities

The liver is a giant “chemical plant” in the human body and plays an important role in the detoxification, storage of glycogen and secretion protein synthesis; it also assumes other functions. The kidney plays a role in maintaining environmental stability in the body. The liver and kidney are important to the human body. With the continuous improvement in living standards, excessive drinking, staying up late, eating food lacking nutrition and other acts detrimental to health are all increasingly common, causing adverse effects on the liver and kidney. Mulberry marc anthocyanins could decrease the contents of alanine aminotransferase, aspartate aminotransferase, hyaluronidase acid, hydroxyproline and collagen type-III in carbon tetrachloride (CCl4)-induced liver fibrosis rats [141]. Mulberry can activate AMPK and PPAR-α signals, reducing the levels of triglyceride (TG), total cholesterol (TC), aspartate aminotransferase (AST), alanine aminotransferase (ALT), and alkaline phosphatase (ALP), enhancing the activity of alcohol dehydrogenase in liver tissue and inhibiting the expression of lipid synthesis-related proteins, thereby preventing liver damage caused by alcohol [111,142]. Mulberry crude extract can promote the expression of Nrf2 in the liver. Nrf2 can induce the downstream phase II detoxification enzyme and phase III transporters, accelerating the metabolism of nonylphenol [42]. Mulberry significantly inhibits blood urea nitrogen (BUN) and Scr and interferes with D-galactose-induced renal cell injury. It can also effectively improve renal function in rats with a certain dose-effect relationship. Mulberry polysaccharide also has a good effect on liver function, which activates alcohol dehydrogenase for subsequent detoxification [143].

### 4.7. Anti-Aging Activity

There are common causes and risk factors for the occurrence and development of human aging and many chronic degenerative diseases such as diabetes, hypertension, Alzheimer’s disease, atherosclerosis and various types of cancer. They are mainly caused by the harmful effects of free radicals (aging factors) on cell components and are the result of oxidation of the blood. The extract of mulberry is rich in phenolic substances and pigments that can significantly reduce the content of β-amyloid protein, increase the activity of antioxidant enzymes and delay memory loss in the aging process [144,145].

### 4.8. Other Effects

Black mulberry extract can inhibit the induction of gamma irradiation and decrease the numbers of micronucleated polychromatic erythrocytes (MnPCEs) and micronucleated normochromatic erythrocytes (MnNCEs) in rat bone marrow cells and increase the ratio of PCE/PCE+NCE, thus reducing the toxic effects on bone marrow cells and the lethal effect of ionizing radiation [100]. Studies have shown that mulberry anthocyanin inhibits pancreatic islet degeneration, which may be associated with autophagy [40]. Jiang et al. found that mulberry can prolong the swimming time of mice and enhance their ability to ward off fatigue, laying an important foundation for mulberry to become a new type of anti-fatigue compound [43]. Mulberry extract is rich in flavonoids, which can lower the activity of tyrosinase, inhibit the production of melanin and treat skin pigmentation disease. This inhibitory effect is closely related to the antioxidant effect of mulberry [146,147]. In addition, mulberry extract can improve depression and relieve convulsions [148].

## 5. Conclusions

Mulberry is rich in nutrition and contains many functional components; it has high edible and medicinal value. The present review has highlighted the nutrients of mulberry fruits, particularly phenolic compounds, which show positive medicinal potential on health. Meanwhile, mulberry’s bioactive phytochemicals might help with a variety of chronic conditions.

Moreover, along with the advances in science and technology, great progress has been made in the study of the active ingredients of mulberry, but the research on the functional composition of mulberry is still insufficient, and it is the key to being able to complete future dietary intervention studies. Therefore, research on the functional components of mulberry should be intensified so that mulberry can be better integrated with industrialization. Additional data would enhance and promote the development and utilization of mulberry resources.

## Figures and Tables

**Table 1:** Total phenol and total flavonoid content in mulberry.

Element	Mulberry	Origin	Concentration	Reference
Total phenolic	*M. alba, M. Nigra, M. rubra*	Olur town, Erzurum, Turkey	181–1422 mg GAE/100g FW	[4]
	*M. nigra* L. and *M. alba* L.	Jinhua, Zhejiang, China	879–6585 mg GAE/kg FW	[12]
	8 different varieties	Orihuela (latitude 38°04′08″ N × longitude 0°58′58″ W, 27 m above sea level) Alicante (South-Eastern Spain)	6.98–13.59 mg GAE/g DW	[13]
	*M. alba* L.	Qinshui County, Shanxi province in China	23.00 mg/g MFP	[15]
	22 different varieties	Quanxi town, Wuyi county of Zhejiang Province, China	199.45–2330.40 μg GAE/g FW	[25]
	4 different varieties	northern regions of Pakistan	880–1650 mg/100 g FW	[27]
	*M. alba* L.	Taichung, Taiwan	1515.9 mg GAE/100 g FM	[31]
	different varieties		7.0–2392.0 mg GAE/100 g	[32]
	*Morus Microphylla Buckl*	Yangpyeong, Korea	24.01 mg/g DW	[33]
	*M. nigra* L.	Istanbul, Turkey	1451.4 mg GAE/100 g DW	[34]
	*M. alba* L.	Silk Innovation Center, Mahasarakham University, Thailand	104.78–213.53 mg GAE/100 g DW	[35]
	*Morus alba* L., *Hongguo no.2*	Shaanxi, China (34°16′–56°24′ N, 108° 4′–27°95′ E)	524.06 mg/100 g DW	[36]
	*M. alba* L.	Suncheon City, Korea	11.2 mg FAE/kg DW	[37]
	10 different varieties	Yinchuan, Ningxia; Zaozhuang (Shandong); Jurong (Jiangsu); Guangzhou (Guangdong)	670–7700 mg GAE/kg FW	[38]
	*M. alba* L.	National Institute of Agricultural Science and Technology, Suwon, Korea	959.9–2570.4 μg GAE/g dried extracts	[39]
	*M. alba* L.	Hangzhou, China	547.60 mg GAE/g MAE	[40]
	*M. nigra* L.	Yesilyurt, Malatya (38.321059, 38.217478)	192.67 mg GAE/g	[41]
		Guangzhou, Guangdong, China	35.53% in the proportion of dry matter	[42]
	*M. alba L.*	Anji and Fuyang, Zhejiang, China	11.67–690.83 mg GAE/g	[43]
	Dried mulberry fruits juice *(Morus* sp.)	Xinjiang, China	3.21 mg GAE/g	[44]
	*M. nigra* L.	Puerto Real region (Spain)	1301.67 μg/g FW	[45]
	11 different varieties	Zhejiang province, China	100.97–586.23 mg GAE/100 g FW	[46]
	*M. nigra* L.	Ordu, Turkey	2032.87 mg GAE/100 g DW	[47]
	Mulberry fruit	Sang-ju Silkworm Farming Association, Sang-ju, Korea	5.16 mg/100 g	[48]
	*M. alba* L.	Jinhua, Zhejiang, China	185–344 mg 100/g FW	[49]
	*M. nigra* and *M. rubra*	Turkey	1005–3488 μg GAE/g FW	[50]
	*M. nigra* L.		1375 mg GAE/100 g DW	[51]
Total flavonoids	*M alba, M. nigra, M. rubra*	Olur town, Erzurum, Turkey	29–276 mg QE/100 g FW	[4]
	*M. nigra* L. and *M. alba* L.	Jinhua, Zhejiang, China	663–1292 mg QE/kg FW	[12]
	*M. alba* L.	Qinshui County, Shanxi, China	3.90 mg/g MFP	[15]
	*M. alba* L.	Taichung, Taiwan	250.1 mg QE/100 g FM	[31]
	*Morus alba* L., *Hongguo no.2*	Shaanxi, China (34°16′–56°24′ N, 108° 4′–27°95′ E)	463.62 mg/100 g DW	[36]
	38 different varieties	all around China	0.178–2.485 mg RE/g lyophilized mulberry fruit	[52]
	*M. alba* L.	National Institute of Agricultural Science and Technology, Suwon, Korea	5.6–65.4 μg/g DW	[39]
	*M. alba* L.	Hangzhou, China	893.73 mg RE/g mulberry anthocyanin extract	[40]
	*M. nigra* L.	Yesilyurt, Malatya (38.321059, 38.217478)	125.86 mg QE/g	[41]
		Guangzhou, Guangdong, China	7.53% in the proportion of dry matter	[42]
	*M. alba* L.	Anji and Fuyang, Zhejiang, China	94.53–965.63 mg RE/g	[43]
	*ML juice*	Xinjiang, China	53.85 mg QE/g	[44]
	11 different varieties	Zhejiang province, China	16.38–368.16 mg RE/100g FW	[46]
	*Mulberry fruit*	Sang-ju Silkworm Farming Association, Sang-ju, Korea	9.73 mg/100g	[48]
	*M. nigra* L.		1473 mg RE/100g DW	[51]

FW: fresh weight; DW: dry weight; MAE: mulberry anthocyanin extract; RE: rutin equivalents; GAE: gallic acid equivalents; FAE: ferulic acid equivalents; FM: fresh matter; QE: equivalent of quercetin; MFP: powder of mulberry (*Morus alba* L.) fruit (MFP).

**Table 2:** Anthocyanin in mulberry.

Mulberry	Anthocyanin	Origin	C3G	C3R	Reference
4 different varieties	184.3–227.0 mg/100g	Van province			[3]
*M. alba* L.		Jinhua, Zhejiang, China	1698 mg/kg FW	693 mg/kg FW	[12]
*M. alba* L.	0.01–1.88 mg/g DW	Orihuela (latitude 38°04′08″ N × longitude 0°58′58″ W, 27 m above sea level) Alicante (South-Eastern Spain)	0.004 –1.26 mg/g DW	0.004–0.08 mg/g DW	[13]
*M. alba* L.	0.87 mg/g	Qinshui County, Shanxi, China			[15]
22 different varieties	306.91–1422.11μg/g	Quanxi town, Wuyi county of Zhejiang Province, China			[25]
*Morus Microphylla Buckl.*	2.3 mg/g DW	Yangpyeong, Korea			[33]
different varieties		Zhejiang, China	1.25–3.35 g/kg	0.25–1.50 g/kg	[56]
*Morus nigra* L.		Istanbul, Turkey	1221 mg/100 g DW		[34]
41 different varieties	0.87–96.08 mg/g lyophilized mulberry fruit	all around China	0.57–62.93 mg/g lyophilized mulberry fruit	0.05–12.70 mg/g	[52]
*M. alba* L.	137.3–2057.3 μg/g	National Institute of Agricultural Science and Technology, Suwon, Korea	93.2–1364.9 μg/g DW	30.6–486.7 μg/g DW	[39]
*M. alba* L.	77.9% of the whole extract	Hangzhou, China			[40]
*M. alba* L.	24.10–383.49 mg/g	Anji and Fuyang, Zhejiang, China	19.30–272.00 mg/g DW		[43]
11 different varieties	4.20–121.56 mg catechin equivalents/100 g FW	Zhejiang province, China			[46]
*M. nigra* L.		Ordu, Turkey	1572.41 mg/100 g DW		[47]
*Mulberry fruits*		Sang-ju Silkworm Farming Association, Sang-ju, Korea	97.68 mg/100 g	71.18 mg/100 g	[48]
*M. nigra* L., *M. rubra* L.	3–830 μg/g	Turkey			[50]
*M. alba* L.	669 mg/100g DW		371 mg/100g DW		[51]
*M. alba* L.		São Paulo city, Brazil	79% of anthocyanin	19% of anthocyanin	[62]
	0.19–3.29 mg/g				[63]
31 kinds cultivated mulberry juices	147.68–2725.46 mg/L	Quanxi town, Wuyi county of Zhejiang Province, China			[64]
180 different varieties	0.035–2.192 mg/g	Huzhou Academy of Agricultural Sciences, China			[65]

FW: fresh weight; DW: dry weight.

**Table 3:** The healthy effects of anthocyanins in mulberry.

Anti-Oxidant						
Mulberry	Anthocyanins	Duration of Study	Models	Method	Effect	Reference
10 mulberry cultivars	C3G and P3G			HPLC-QTOF-MS	↓ α-glucosidase	[38]
*Morus alba* L.	C3G and C3R		Human hepatoma cell HepG2	HPL cytotoxicity assay C, Western blot analysis	↓ acetyl coenzyme A carboxylase activities ↑ the lipolytic enzyme expressions of PPARα and CPT1	[96]
Fresh mulberry		16-week	Male C57BL/6 mice 4 weeks old	Animal experiment with high-fat diet, Enzyme-linked immunosorbent assay (ELISA), RT-PCR	↓ MDA production ↑ SOD and GP.sub.X activities	[99]
*Morus alba* L.	C3G, C3R and P3G	Animal experiments: 10-week	HepG2 cells, Male db/db mice	Histology and immunohistochemistry analysis, Western blot analysis, transmission electron microscopy	↓ islet degeneration, which may be due to autophagy stimulation ↓ impaired mitochondria dysfunction ↓ excessive free radical production	[40]
*Morus alba* L.	C3G			Total reducing power assay (TRP assay), DPPH assay, ferric reducing antioxidant power (FRAP assay)	↓ DPPH free radical ↓ stress-induced oxidative damage	[43]
11 mulberry cultivars			Human intestinal epithelial cell line Caco-2	HPLC-TOF-MS, Determination of cellular ROS, Ccll culture	↑ ROS scavenging activity	[46]
*Morus alba* L.	cyanidin 3-O-(6″-O-α-rhamnopyranosyl-β-d-glucopyranoside) (C3RG), cyanidin 3-O-(6″-O-α-rhamnopyranosyl-β-d-galactopyranoside) (C3RGa), cyanidin 3-O-β-d-glucopyranoside (C3G), cyanidin 3-O-β-d-galactopyranoside (C3Ga) and cyanidin 7-O-β-d-glucopyranoside (C7G)			HPLC, ESI-MS, nuclear magnetic resonance (NMR)	↓ DPPH free radical	[104]
*Morus alba* L.		2-week	Male Kunming mice (18–20 g)	HPLC-PDA analysis, spectrophotography	↓ DPPH free radical and superoxide anion radicals ↑ antioxidant enzymatic activities (SOD, CAT, GSH-Px)	[105]
**Anti-diabetic**						
*Morus alba* L.	C3G and C3R	5-week	Male C57BL/KsJ-db/db mice 5 weeks old	Insulin tolerance test, HPLC, the radioimmunoassay with an enzyme-linked	↑ AMPK and AS160 in skeletal muscles ↓ gluconeogenesis in the liver ↑ phosphorylated (p)-AMP-activated protein kinase (pAMPK), p-Akt substrate of 160kDa (pAS160) and plasma membrane-glucose transporter 4 (GLUT4) in skeletal muscles ↓ the levels of glucose 6-phosphatase and phosphoenolpyruvate carboxykinase in the liver	[19]
*Morus alba* L.	C3G, C3R, P3G and pelargonidin 3-rutinoside	6-week	Murine macrophage-like cells and rat renal tubular epithelial cells, Five-week-old male ZDF (Lepr fa/CrlCrlj) and age-matched lean rats (Lepr fa/±)	Cell culture, MTT assay, HPLC-ESI-MS/MS, histology and immunohistochemistry analysis	↓ islet degeneration and the progressive decline in insulin secretion ↓ type 2 diabetes	[106]
*Mulberry*	C3G		MIN6N pancreatic β-cells (derived from a mouse pancreatic islet)	Cell culture, MTT assay, immunofluorescent staining, flow cytometric and Western blot analyses	↓ intracellular reactive oxygen species, DNA fragmentation and the rate of apoptosis ↓ pancreatic β -cell apoptosis induced by high glucose conditions ↑ insulin secretion	[107]
*Morus alba* L.		Animal experiment: 8-week	HepG2 cells, Four-week-old male C57BL6/J genetic background (db/db) mice and their nondiabetic lean littermates (m/m)	Cell culture, histology and immunohistochemistry analysis, MTT assay, Western blot analysis, RT-PCR	In vitro: ↓ insulin resistance, ↓ PGC-1α and forkhead box protein O1 (FOXO1), enzyme activities of phosphoenolpyruvate carboxykinase (PEPCK) and glucose-6-phosphatase (G6Pase) ↑ glucose consumption, glucose uptake and glycogen content In vivo: ↓ fasting blood glucose, serum insulin, leptin, triglyceride and cholesterol levels ↑ adiponectin levels	[108]
*Morus alba* L.		2-week	Male Kunming mice (18–20 g)	HPLC-PDA analysis, spectrophotography	↓ fasting blood glucose, glycosylated serum Protein and anti-α-glucosidase	[105]
*Morus alba* L.			Human non-tumor hepatic cell line, LO2, C. elegans maintenance	Glucose consumption and uptake assays, ROS, O_2_^−^, mitochondrial membrane potential (MMP) and mitochondrial numbers assays, Western blot, RNA isolation and qPCR analysis	alleviate cellular damage and this effect is related to Nrf2 ↑ the life span of C. elegans ↓ lipid peroxidation accumulation ↑ SOD and GPx activity and PMK-1 expression	[109]
**Anti-inflammatory**						
*Morus alba* L.			Peritoneal macrophages (Female BALB/c strain mice (10 weeks old) weighing 20–25 g)	HPLC, ELISA, cell culture, ELISA, MTT	↓ splenocytes’ (IFN-γ + IL-2 + IL-12)/IL-10 (Th1/Th2) cytokine secretion ratios and TNF-α/IL-10 (pro-/anti-inflammatory) cytokine secretion ratios	[67]
Fresh mulberry		16-week	Male C57BL/6 mice at 4 weeks of age	Animal experiment with high-fat diet, ELISA, RT-PCR	↓ TNFα, IL-6, iNOS and NF-κB	[99]
*Morus alba* L.	C3G, C3R and P3G	Animal experiments: 10-week	HepG2 cells, Male db/db mice (C57BL6/J genetic background, 4 weeks of age) and their nondiabetic lean littermates (m/m)	Histology and immunohistochemistry analysis, Western blot analysis, transmission electron microscopy	↓ the epididymal adipose mRNA expression of PPARγ, IL-6 and IL-1β ↑ the expression of SREBP-1c and C/EBP	[40]
*Morus alba*			Peritoneal macrophages, 6 weeks old female BALB/cByJNarl mice	Cell culture, MTT, ELISA	Mulberry juice (10–500 μg/mL): ↓ pro-inflammatory cytokines TNF-a secretions by LPS-stimulated peritoneal macrophages ↑ the secretion of anti-inflammatory cytokine IL-10 Mulberry juice (10μg/mL): ↑ TNF-a Mulberry juice (500μg/mL): ↑ IL-10	[110]
*Morus alba* L.		6-week	Seven-week-old male C57BL/6J mice weighing 20 g	HPLC, histology and immunohistochemistry analysis, Western blot analysis	↓ iNOS, COX2, NF-κB, TNF- a and IL-6	[111]
**Anti-cancer**						
*Morus alba* L.	C3G and C3R		A549, a human lung cancer cell line obtained from ATCC (Manassas, VA, USA)	MTT assay, cell migration and invasion assays	↓ matrix matalloprotinase-2 and urokinase-plasminogen activator (u-PA) ↑ the expression of tissue inhibitor of matrix matalloprotinase-2 (TIMP-2) and plasminogen activator inhibitor (PAI)	[93]
Mulberry Fruit (*Moris fructus*)			A172 cells	Immunohistochemistry analysis, Western blot analysis	↓ tumor cell proliferation ↑ tumor cell apoptosis	[112]
*Morus alba* L.		Dextran sulfate sodium (DSS)-induced acute colitis model: 19-day; MUC2^−/−^ mouse model: 3-month	RAW 264.7 macrophages, Six to eight-week-old BALB/c mice, and MUC2 ^−/−^ mice with colorectal cancer	Cell culture, real-time quantitative PCR, Western blot analysis, ELISA, Animal disease models and diets	↓ proinflammatory mediators and cytokines (iNOS, COX-2, IL-1β and IL-6) ↓ prevent NF-κB/p65 and MAPK/pERK signals	[113]
*Morus alba* L.		Animal experiments: 5-week	C57BL/6 mice, B16-F1 (a murine melanoma cell line)	Western blot analysis HPLC, MTT assay, Wound-healing assay, Boyden Chamber migration assay	↓ NF-κB transcriptional factor ↓ DNA binding activity of NF-κB to the NF-κB response element ↓ MMP-2 and MMP-9 activities	[114]
*Morus alba* L.			SW1736 (BRAFV600^E/wt^) and HTh-7 (NRAS^Q61R^) thyroid cancer cells	MTT colorimetric assays, cell migration and invasion assays, Colony formation assay, GFP-LC3 transient transfection, flow cytometry assay, Western blot analysis	↑ apoptosis and autophagy ↓ thyroid cancer cell proliferation and Akt/mTOR signaling	[115]
Lyophilized fruit of Mulberry	C3G and C3R	Animal experiments: 49-day	AGS cell line (obtained from the Bioresource Collection and Research Center), Balb/c nude mice (male, 5 weeks old)	Western blotting analysis, HPLC	↑ intrinsic and extrinsic apoptosis through p38/p53 and p38/c-jun signaling pathways	[116]
Mulberry juice		24-h	BALB/c mice	Animal experiment	↓ the growth of Porphyromonas gingivalis, Prevotella melaninogenica	[117]
**Anti-obesity**						
*Morus alba* L.			3T3-L1 preadipocyte cells (mouse embryonic fibroblast-adipose like cell line)	GC–MS, cell culture, Western blot analysis, TUNEL assay	↑ mitochondrial dysfunction, DNA Fragmentation and cell apoptosis ↓ the proliferation of 3T3-L1 preadipocyte cells	[19]
*Morus alba* L.	C3G and C3R		human hepatoma cell HepG2	HPL cytotoxicity assay C, Western blot analysis	↓ fatty acid synthesis ↑ fatty acid oxidation ↓ the expression of sterol regulatory element-binding protein-1 and its target molecules	[96]
Fresh mulberry		16-week	The male C57BL/6 mice with 4 weeks of age	Animal experiment with high-fat diet, Enzyme-linked immunosorbent assay (ELISA), RT-PCR	↓ serum glucose and leptin levels	[99]
*Morus australis* Poir	C3G, C3R and P3G	12-week	The male C57BL/6 mice with 4 weeks of age	Animal experiment with high-fat diet (HFD)	↓ insulin resistance, the size of adipocytes, lipid accumulation and leptin secretion	[118]
*Morus alba* L.	C3G, C3R, P3G and pelargoni-dine-3-rutinoside	12-week	6-week-old male hamsters	HPLC/ESI-MS-MS	↓ serum triacylglycerol, cholesterol, free fatty acid, the LDL/HDL ratio ↑ the hepatic peroxisome proliferator-activated receptor R and carnitine palmitoyltransferase-1 ↓ fatty acid synthase and 3-hydroxy-3-methylglutaryl-coenzyme A (HMG-CoA) reductase ↓ the ratio of gonadal fat and pararenal fat	[119]
Mulberry fruit	C3G, C3R		Mouse 3T3-L1 cells,	qRT-PCR, assay kit, cell culture	↓ intracellular lipid content, TG, the expression of adipogenic genes in adipocytes which may be associated with AMPK activation	[48]
Dried mulberry fruit powder obtained from *M. alba* L.		Animal experiments:3 months	3T3-L1 preadipocytes; female C57BL/6J mice with 8 weeks of age	LC-MS, Animal experiment with high-fat diet (HFD), histological analysis, biochemical analyses,	↓ TG, TC/HDLC, cell fat ↑ HDLC	[120]
*Morus alba* L.		10-week	Thirty male New Zealand white rabbits, weighing 2000–2200 g	Animal experiment with high-cholesterol diet (HCD)	↓ serum cholesterol and triglyceride and repress progression of atherosclerosis	[121]
*Morus alba*		12-week	Male C57BL/6 J mice (3 weeks of age)	Histological analysis, biochemical analyses, enzyme activity analyses, qRT-PCR	↓ insulin and glucose levels ↓ plasma lipid levels and lipid accumulation in the liver ↑ the activities of hepatic fatty acid b-oxidation enzymes (CPT and ACO), Ppara mRNA expression and plasma adiponectin level	[122]

↑ : increase of substance; ↓ : decrease of substance.

**Table 4:** Anti-oxidant activity of mulberry.

	Mulberry	Concentration	Reference
DPPH	*M. alba* L. and *M. nigra* L.	0.52–6.43 mg VCE/g	[12]
	*M. nigra* L.	0.0362–0.1291 mg TE/100 g	[13]
	22 different varieties	4.41–508.08 mg TE/100 g	[25]
	*M. alba* L. and *M. nigra* L.	10.7–14.5 mg TE/100 g	[26]
	*M. alba* L.	29.19–44.71 mg TE/100 g	[68]
	*M. alba* L.	22.01–698.57 mg TE/g	[43]
	*M. nigra* L.	946 mg TE/100 g	[51]
	8 different varieties	2.5–20.3 µmol TE/g	[123]
ABTS	four different varieties	0.44–1.39 mg TE/100 g	[3]
	*M. nigra* L.	0.0384–0.2073 mg TE/100 g	[13]
	22 different varieties	33.57–438.25 mg Ascorbic acid/100 g	[25]
	*Eleven mulberry cultivars*	217.01–850.85 mg VCE/100 g FW	[45]
	*M. nigra* L.	2788 mg TE/100 g	[51]
	*M. nigra* L. and *M. rubra* L.	0.51–1.44 mg TE/100 g	[50]
FRAP	22 different varieties	0.26–4.87 mmol Fe^2+^/100 g	[25]
	*Eleven mulberry cultivars*	11.92–319.40 mg VCE/100 g FW	[46]
	*M. nigra* L. and *M. rubra* L.	0.37–1.69 mg TE/100 g	[50]
	*M. nigra* L.	1836 mg TE/100 g	[51]
OH	22 different varieties	33.57–438.25 mg Ascorbic acid/100 g	[25]
ORAC	*M. rubra*	0.301–1.728 mmol TE/g	[124]
CUPRAC	*M. nigra* L.	4046 mg TE/100 g	[51]

DPPH: 1,1-diphenyl-2-picrylhydrazyl; ABTS: 2,2′-azino-bis (3-ethylbenzothiazoline-6-sulfonic acid); FRAP: ferric reducing antioxidant power; OH: hydroxyl radicals; ORAC: oxygen radical absorbance capacity; CUPRAC: copper reducing antioxidant capacity; TE: Trolox equivalents, VCE: vitamin C equivalents; FW: fresh weight.

## Data Availability

Not applicable.
